# Prevalence, associated factors and treatment of post spinal shivering in a Sub-Saharan tertiary hospital: a prospective observational study

**DOI:** 10.1186/s12871-016-0268-0

**Published:** 2016-10-18

**Authors:** Tonny Stone Luggya, Richard Nicholas Kabuye, Cephas Mijumbi, Joseph Bahe Tindimwebwa, Andrew Kintu

**Affiliations:** 1 Department of Anaesthesia, School of Medicine, College of Health Sciences, Makerere University, Kampala, Uganda; 2 Directorate of Surgery, Mulago National Referral Hospital, Kampala, Uganda

**Keywords:** Post spinal shivering, Caesarean section, Spinal anaesthesia, Pethidine (meperidine)

## Abstract

**Background:**

Surgery and anaesthesia cause shivering due to thermal dysregulation as a compensatory mechanism and is worsened by vasodilatation from spinal anaesthesia that redistributes core body heat. Due to paucity of data Mulago Hospital’s post spinal shivering burden is unknown yet it causes discomfort and morbidity.

**Methods:**

Ethical approval was obtained to perform the study among consenting mothers due for elective caesarean section from March to May 2011. We recruited ASA class I & II parturients and excluded non-consenting or spinal contra-indication patients. A standard spinal anaesthetic of 2mls of 0.5 % bupivacaine was given, intraoperative vitals were recorded every 5 min and we monitored for perioperative shivering till PACU discharge.

**Results:**

We recruited 270 patients with majority being emergency caesarean deliveries (90.74 %), mainly due to failed progress from cephalopelvic disproportion. We noted 8.15 % shivering occuring mostly at 20 min, with hypotension plus hypothermia as associated factors. Intravenous pethidine (Meperidine) 25 mg effectively treated shivering and we had drowsiness, nausea and vomiting as PACU side effects that resolved on discharge to the ward.

**Conclusion:**

Post spinal shivering had a prevalence of 8.15 %, commonly occurred at 20 min postoperatively with hypotension plus hypothermia as main associated factors and intravenous Pethidine controlled it.

## Background

The autonomic nervous system, by a combination of physiologic and behavioural changes, maintains core temperature between 36.5 and 37.5 °C despite external environmental temperature changes [1], while anaesthesia causes a phase like decline of core temperature with phase I-the greatest- occurring at 30mins, phase 2 after 1 h and then phase 3 after 3–5 h with reduced heat loss till equilibrium is reached [2, 3]. Surgery causes heat loss due to exposure to cold environments, evaporation from exposed sites and administration of unwarmed fluids leading to Core hypothermia that causes shivering as a compensation mechanism. Hypothermia leads to postoperative shivering, prolonged hospital stay, surgical wound infection, decreased immunity and coagulopathy, and increased incidence of cardiac morbidity [4, 5].

Post spinal shivering is an unpleasant, thoroughly discomforting and frequent complication after surgery with many grades i.e. from a mild form of having skin eruptions to a severe form with generalised continuous skeletal muscle contractions with prevalence of up to 50–80 % [6]. Exact causes of post spinal shivering are still unclear though various mechanisms have been postulated with some attributing it to a thermoregulatory response to hypothermia that causes temperature-induced changes of neurons in the mesencephalic reticular formation and dorsolateral pontine and medullary reticular formation [7]. It is an involuntary, oscillatory muscular activity that augments metabolic heat production up to 600 % above basal metabolic level [8] and clinically is associated with clonic or tonic skeletal muscle hyperactivity of different frequencies [9]. This increased muscular activity leads to increased oxygen consumption and carbon dioxide production that results in hypoxaemia, hypercarbia and lactic acidosis which are not only discomforting but also worsens pain sensation [6]. Shivering can be prevented by maintaining intraoperative normothermia, giving warm fluids, using warm clothing covers sites or by administering pharmacologic treatments like tramadol, clonidine and pethidine (meperidine) [10–15]. Mulago National Referral and Teaching Hospital (MNRTH) hospital is faced with inadequate staffing and overwhelming patient numbers with a nurse: patient ratio of 1: 40 [16], coupled with essential drug shortages where 45 % anaesthesia providers only either having either pethidine or morphine and 21 % never have these drugs available for perioperative patient management [17]. We thus sought to determine the prevalence, associated factors and effect of intravenous pethidine (meperidine) on post spinal shivering among mothers undergoing spinal anaesthesia for caesarean section delivery at MNRTH.

## Methods

This study was approved by the Makerere University, School of Medicine Research and Ethics Committee (SOMREC) and we conducted a descriptive cross-sectional study among parturients in MNRTH from March 2011 to May 2011. Mulago is Makerere University College of Health Sciences teaching hospital offering training to post graduate and undergraduate medical students, anaesthetic officers, midwives and a whole range of other health workers. It has a bed capacity of 1500 beds that increase during annual epidemics. It serves a national population of over 33,000,000 people and has 13 operating theatres with 24 operating tables that conduct over 8000 operations per annum. The labour suit carries out 32,000 deliveries annually with caesarean sections accounting for 15–20 %. We recruited consenting mothers with American Society of Anaesthesia (ASA) class I and II scheduled for caesarean section in the study period and we excluded patients that declined to consent, had allergies to study drugs or contra-indications to spinal anaesthesia like i.e. suspected placenta Previa haemorrhage, expected excessive haemorrhage from ruptured uterus, skin infection at the back, etc.

### Procedurally

Under aseptic conditions all study patients included in our study received the standard care at MNRTH which was spinal anaesthetic of bupivacaine 2 ml of 0.5 %, oxygen by nasal prongs at the rate of 2 l/min and were placed supine with left lateral tilt position until bilateral T6 block was achieved for surgery to commence. All parturients axilla temperature was measured and they got standard warmed fluids with ambient operating room temperature kept between 21^0^ and 25 °C by air conditioning. In the Post Anaesthesia Care Unit (PACU) the patients’ vitals (BP, temperature, heart rate, MAP and respiratory rates) were recorded at 5 min intervals for the first 30 min then every 15 min.

All patients included in your study received standard care and no changes to care were made as a result of our study.

### Study variables

Main study outcome was shivering with the main independent variable was time to shivering; other independent factors considered included hypotension and hypothermia following the spinal anaesthesia which when got was concomitantly treated and if both occurred with shivering was treated first, according to MNRTH protocols, before giving intravenous pethidine 25 mg. We graded Shivering using Crossley and Mahajan scale [18] as shown in Appendix and treated it with 25 mg of intravenous pethidine (meperidine). All mothers were followed up for 24 h.

### Sample size calculation

The proportion of shivering was determined using the 2009 study by Javaherforoosh et’al [9] and using Kish and Leslie formula with 95 % confidence, with a 50 % chosen precision and Z being 1.96 plus factoring in loss to follow up (5 %) we derived a sample size of 173 .

### Data collection and analysis

Data was collected using interviewer administered, pre-coded, pre-tested and standardized questionnaire. It was cleaned, coded entered with EPI-DATA version 2.0 and analysed it in STATA 10. The distributions of study participant baseline characteristics were presented as frequencies with respective proportions and results were reported using proportions, means, medians and inter-quartile ranges. Univariate analysis was done for the proportion of subjects experiencing Post spinal shivering with estimation of the odds of the difference different shivering categories. Chi-squared test was used to determine associations between predictor and each outcome variables with *p*-value < 0.05 considered as statistically significant in all analysis.

## Results

### Participants’ characteristics

**Fig. 1:**
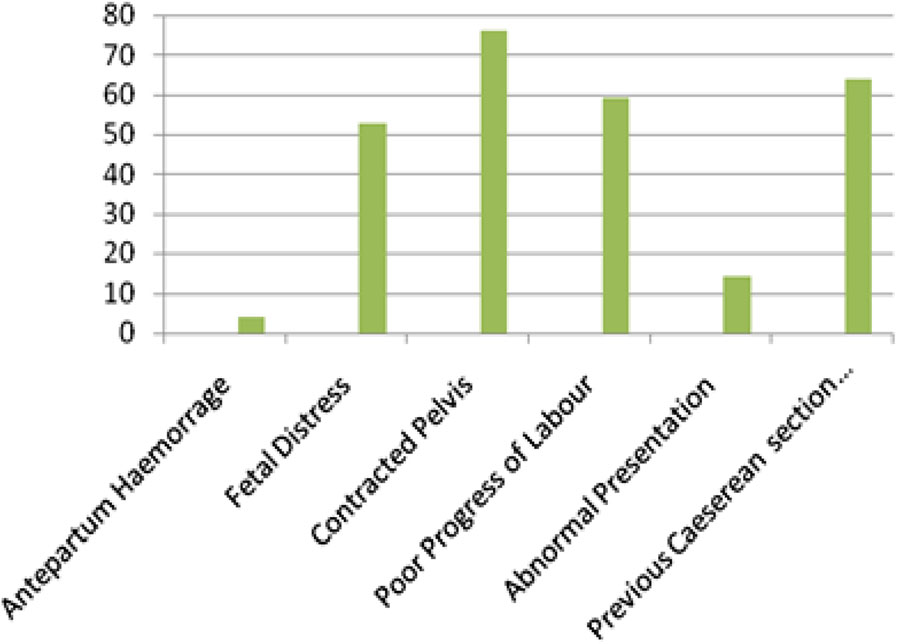
Bar graph showing indications for C/S

We screened 346, enrolled 270 mothers and excluded 76 with an average mother’s age of 25. Clinically the Mean Systolic Blood Pressure was 132.7, mean MAP was 93.2 and Heart Rate = 103. Emergencies’ accounted for 90.74 % with the commonest indication being contracted pelvis (28.15 %) and least indication being antepartum haemorrage (1.48 %) as shown in Fig. 1.

### Shivering and factors associated

**Fig. 2:**
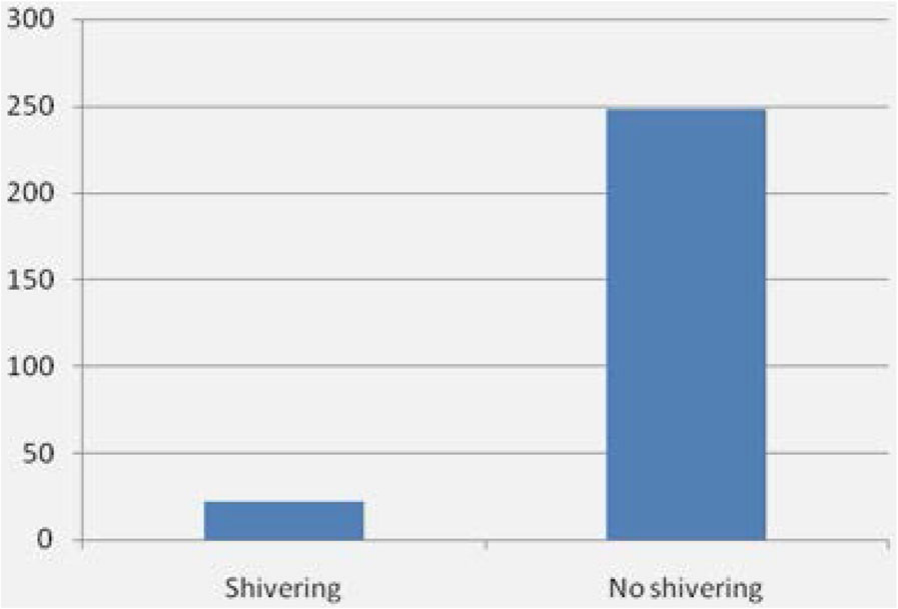
Bar graph showing prevalence of shivering

Shivering was witnessed in 22 patients with a prevalence of 8.15 %, as shown in Fig. 2, of which majority(16) were gradeI observations and the reset (6) were grade 2 with no observation for grade 3 & 4.

Mean time of shivering was between 15 to 25 min with majority of the shivering occurrying at 20 min as shown in Table 1.

**Table 1:** Showing shivering times

Time in minutes	Number of Observations of Shivering
5	1
10	7
15	11
20	16
25	10
30	4

Patients that got shivering were given 25 mg pethidine intravenously that effectively alleviated with most subsiding to grade0 (no shivering) within 5 min.

Hypotension and hypothermia were the main factors associated with shivering among mothers in this study with mothers mean body temperature was36.6+/− 1 °C and mean OR temperature kept at 27.4+/− 0.85 °C as shown in Table 2.

**Table 2:** Room and patient mean temperatures

Associated factor	Recording
Mean temperature of the room	27.4 +/− 0.85 °C
Mean temperature of the Patient	36.6 +/− 1 °C
Mean temperature difference	9.27 +/− 1.3

Side effects noted among those that received pethidine were *arousabl*e drowsiness; Nausea and Vomiting, which on analysis had insignificant *p*-values (Table 3) and, clinically were not noted at PACU discharge.

**Table 3:** Common side effects of pethidine

Side effect	Affected patient number (*n*)	*P*-value
Nausea	3 (8.6)	0.922
Vomiting	3 (8.6)	0.985
Drowsiness	6 (17)	0.89

## Discussion

This study was done to determine the prevalence of shivering, its associated factors and effect of treatment with pethidine (meperidine). We noted an 8.15 % prevalence of post spinal shivering with intraoperative hypotension plus hypothermia as main associated factors. Intravenous pethidine (25 mg) by 5 min had adequately treated shivering mong mothers we studied. We did this study because post-operative shivering in MNRTH is over looked yet it causes significant discomfort and also studies have ranked it 8^th^ as a complication, and 21^st^ as easily preventable among post-operative complications [19]. Shivering increases work of various muscle groups including the myocardium causing an increased lactic acidosis plus carbon dioxide production that contribute to wound pain, increased intraocular and intracranial pressure as a result of increased oxygen consumption [9]. Post spinal shivering increases the body’s basal metabolic oxygen demand due to raised oxygen consumption by about 200 to 500 % [20, 21] which in patients with already limited myocardial oxygen supply, e.g. arteriosclerosis, may lead to compromised myocardial function, worsening morbidity as a result of increased vascular resistance from vasoconstriction which in combination with heat and carbon dioxide production due to hypothermia is oxygen draining [22, 23].

Many drugs are postulated to treat or prevent post-operative shivering i.e. pethidine (meperidine) [24, 25], clonidine [14, 26], ketanserin [26], amitriptyline [27], tramadol [9, 27], midazolam [28], magnesium sulphate [29], ondansetron [24] and ketamine [30]. We chose pethidine (meperidine) as it is relatively available option in our setting and studies have shown that pethidine (meperidine) has a more prominent effect on prevention and treatment of postoperative shivering in comparison with other opioids because it’s both a μ- and k-receptor antagonist, unlike μ-receptor agonists (morphine, fentanyl, sufentanil). This we thought held true as the non-opioid effects of meperidine are associated with its anti-shivering action, such as monoamine reuptake inhibition, NMDA receptor antagonism, and stimulation of α-2 receptors [31], thus giving two pronged benefit to our mothers which was shown in this study findings where 25 mg of pethidine effectively controlled post spinal shivering. In light of the immediate nausea, vomiting and arousable drowsiness side effects of intravenous pethidine we recommend further studies on prevention of shivering with probable intrathecal low dose pethidine as studies have shown its benefit in alleviating the risk of inducing nausea and vomiting when given intrathecally [32, 33] albeit with caution due to the risk of foetal bradycardia. Despite routine warming of preoperative fluid In MNRTH as a standard protocol we got hypothermia as a side effect because generally Neuraxial anaesthesia causes an estimated fall of 0.5–1 °C coupled with other contributing factors like patient’s pre-neuraxial temperature, the ambient room temperature and temperature of infused fluids [27, 34]. Fluid warming in general surgical populations is a low cost measure with proven deleterious effects of hypothermia and is also internationally recommended of emergency obstetric haemorrhage [35]. However recent studies have shown there is no reduction in the incidence of shivering with fluid warming and less variation in maternal temperature after caesarean section [36, 37] which strengthens the postulation of shivering occurring due to spinal anaesthesia. Our study limitation was the use of auxiliary temperature and not core temperature this was thought to be a reliable surrogate for core body temperature in our settings though studies have shown it not to correlate in extreme body temperatures [38].

## Conclusion

Post spinal shivering in Mulago National Referral and Teaching Hospital had a prevalence of 8.15 %, occurrying commonly between 15 and 20 min post opratively with hypotension and hypothermia as the main associated factors. We noted intravenous pethidine (25 mg) effectively controlled shivering.

## Appendix

**Table 4:** Crossley and Mahajan post anaesthetic shivering score (pas) used

Grade	Shivering intensity
0	No Shivering
1	Peripheral vasoconstriction associated with sensation of coldness, piloerection without any visible shivering.
2	Visible muscular contraction confined to one muscle group
3	Visible muscular contraction in more than one muscles but not generalised.

## Abbreviations

ASA: American Society Of Anaesthesia
MAP: Mean arterial pressure
MNRTH: Mulago National Referral and Teaching Hospital
PACU: Post Anaesthesia Care Unit
